# Emotional distress among medical students before and after the first exposure to a full cadaver dissection: a quantitative study using DASS-21

**DOI:** 10.1080/10872981.2025.2601707

**Published:** 2025-12-12

**Authors:** Miral Nagy Fahmy Salama, Ahmed Mohamed Abdelkhalek, Mayssah Ahmed El Nayal, Ramya Rathan, Mona Ghazi Sayegh, Marwa Mady

**Affiliations:** a Gulf Medical University, Biomedical Sciences Department, Ajman, United Arab Emirates; b Alexandria University, Faculty of Arts, Psychology Department, Alexandria, Egypt; c Lebanese International University, Psychology Department, Beirut, Lebanon; d Alexandria University, Faculty of Medicine Human Anatomy and Embryology Alexandria, Alexandria, Egypt

**Keywords:** Cadaver dissection, emotional distress, medical students, DASS-21, anxiety, stress, depression, anatomy education

## Abstract

**Background:**

Cadaver dissection remains a foundational component of anatomy education in many medical curricula. While it provides irreplaceable hands-on learning, it may also elicit emotional distress, especially during initial exposures. This study aimed to assess the levels of emotional distress among medical students before and after their first full cadaver dissection using the Depression, Anxiety, and Stress Scale (DASS-21).

**Methods:**

This quantitative, comparative, cross-sectional study explores emotional distress among first-year medical students Higher Diploma in Pre-Clinical Sciences program (HDPCS) at Gulf Medical University -Ajman, UAE. Participants completed the DASS-21 questionnaire immediately before and after their first exposure to full cadaver dissection. Changes in depression, anxiety, and stress scores were analyzed using statistical tests.

**Results:**

A total of 80 students participated in the study. Pre-dissection scores indicated mild to moderate levels of anxiety and stress in a significant proportion of students. Post-dissection assessments showed a statistically significant reduction in anxiety scores (*p* < 0.05), while stress and depression scores showed marginal or no significant change.

**Conclusion:**

The first encounter with a cadaver can provoke acute emotional responses in medical students, particularly anxiety. However, emotional adaptation appears to occur rapidly post-exposure. These findings underscore the importance of psychological preparedness, reflective practices, and institutional support to ensure student well-being during cadaveric dissection sessions.

## Introduction

Students at medical colleges receive their first practical introduction to human anatomy in the dissection room, a crucial component of medical education. However, this exposure may be emotionally demanding, resulting in elevated emotional distress such as stress, anxiety, and depression. These psychological reactions are influenced by the anticipation of handling cadavers, environmental unfamiliarity, and the moral dilemmas surrounding human dissection. Research indicates that students’ learning effectiveness and long-term professional adaptation may be impacted by the emotional distress experienced during early cadaver encounters [1,2]. Furthermore, students' emotional distress differs in intensity according to their cultural background, coping strategies, and previous exposure to anatomy instruction [2].

Emotional distress is a broad term that can refer to a wide range of symptoms stemming from various mental health disorders. However, anyone can experience emotional distress even without meeting the criteria for a specific psychological disorder [3]. It is a temporary state of emotional discomfort or unease that individuals may experience when facing stressful or unfamiliar situations. For medical students, the first visit to the dissection room can cause emotional distress due to the unfamiliar environment, the smell of chemicals like formalin, and the experience of working with human cadavers. This reaction is common and may include feelings of anxiety, fear, sadness, or physical symptoms such as nausea and a rapid heartbeat. It is not considered a mental illness but rather a normal and expected emotional response to a challenging educational experience [4–6].

It is well-established that about one-third of people will experience depression, anxiety, or stress-related disorders at some point in their lives [7]. Beginning with **depression**, it is characterised by persistent sadness, emotional instability, heightened anxiety, low self-esteem, and sensitivity to stress. It often coexists with anxiety and personality disorders and may lead to maladaptive coping behaviours such as avoidance or substance use [8]. According to the World Health Organisation (WHO), depression is a prevalent mental illness that can affect anyone. It is characterised by a persistently depressed mood, loss of enjoyment, or disinterest in activities. This differs from normal mood swings and day-to-day emotional fluctuations. Sleep disturbances and changes in appetite are common in people who are depressed. They might experience hopelessness about the future, thoughts of death, and low self-esteem. Fatigue and difficulty concentrating are also prevalent. A complex interplay of biological, psychological, and social factors contributes to depression [9].

**Anxiety** is another psychological reaction that can describe medical students’ emotional responses to the cadaver exposure. It involves feelings of worry, nervousness, or unease triggered by exposure to cadavers [5]. The American Psychological Association describes anxiety as ‘an emotion characterised by apprehension and somatic symptoms of tension in which an individual anticipates impending danger, catastrophe, or misfortune’ ([10], p. 65). According to the WHO, 301 million people worldwide suffered from anxiety disorders in 2019, making them the most prevalent mental illness. People with anxiety disorders often experience excessive fear or worry about situations [9]. The DSM-5-TR distinguishes between the two terms: anxiety is the expectation of a future threat, while fear is the emotional reaction to a real or perceived immediate threat. While these states overlap, they also differ. Anxiety is more often linked to muscle tension, hypervigilance, and avoidant behaviours, whereas fear is typically associated with spikes in autonomic arousal, thoughts of danger, and escape behaviours ([11], 216).

The **third** emotional distress under study is **stress**, which is frequently the most significant reaction among medical students in the dissection room. It can have a profound impact on their overall well-being and learning experience. Stress is defined as a physiological and psychological response to an emotionally challenging situation [12]. The APA Dictionary of Psychology defines stress as ‘the physiological or psychological response to internal or external stressors. Stress involves changes affecting nearly every system of the body, influencing how people feel and behave.’ For example, it may be manifested by palpitations, sweating, dry mouth, shortness of breath, fidgeting, accelerated speech, heightened negative emotions, and prolonged fatigue [10], 1036). According to the WHO, stress is a state of anxiety or tension triggered by challenging circumstances. Stress is a normal human response that helps us confront obstacles and dangers in our lives. Everyone experiences stress from time to time, but how we manage it significantly affects our overall well-being. Both the body and the mind are impacted. A small amount of stress is beneficial and can enhance our performance in daily tasks, but excessive stress may lead to both physical and mental health issues. By learning coping mechanisms, we can feel less overwhelmed and support our mental and physical health [9].

Literature shows that the most common reactions of the students to cadaveric dissection include headache, disgust, grief or sadness, and light-headedness [13]. However, responses vary across countries such as the Sultanate of Oman and the Hashemite Kingdom of Jordan. At Sultan Qaboos University, students reported recurring visual images of cadavers (38%) and short-term appetite loss (22.5%). The dissection room’s smell triggered most reactions (91%), followed by fear of infection (62%) [4]. Bataineh et al. [14] found lower reaction rates in Jordan: 28.8% reported fear, 28.9% reported recurring cadaveric images, and 19.3% suffered from palpitations.

Findings from Romania revealed that 34.7% of students reported varying levels of fear during initial dissection sessions. Many experienced anxieties and showed both behavioural and physical responses to specific stimuli, which decreased in the second semester. In the first semester, 57% reported frequent visual encounters with cadavers, decreasing to 44.6% in the second semester. The most common coping mechanisms were rationalisation and emotional detachment [15].

Emotional distress, including depression, anxiety, and stress, can transiently disrupt learning attention, psychomotor performance, and professional reflection in anatomy education [5,16]. By measuring these domains before and after dissection, this study provides evidence of how psychological readiness and exposure influence learning efficacy and professional identity formation in medical students. Reviewer 2—Comment 2.

### Rationale of the study

The present study addresses a notable gap in the literature concerning the psychological impact of cadaveric dissection on medical students, particularly in early exposure contexts within Middle Eastern settings. While previous research has broadly acknowledged emotional distress linked to dissection experiences, limited empirical evidence exists that quantitatively assesses pre- and post-dissection emotional states in a culturally specific cohort. The rationale for this investigation emerges from the urgent need to understand how initial exposure to cadaver dissection contributes to psychological discomfort and how these experiences evolve, particularly in contexts where emotional expression in medical education is often stigmatised.

Furthermore, the dissecting room experience is culturally sensitive in addition to being a psychological or educational milestone. Attitudes regarding death, the human body, and emotional expression differ greatly between cultures, and can have a substantial impact on how pupils internalise and cope with their initial cadaveric contact. In many Middle Eastern, Asian, and African countries, for example, the treatment of human remains is fraught with spiritual, religious, and ethical implications that can exacerbate emotional pain. Cultural norms may also hinder open emotional expression, especially in academic or clinical environments where stoicism is commonly associated with professionalism.

As a result, students may suffer internal conflict between cultural expectations and their true emotional reactions, which can lead to concealed worry, stress, or even guilt. Recognising cadaver dissection as a culturally entrenched event broadens the interpretative lens of this study and corresponds with the larger goal of contextualising emotional distress within psychological and sociocultural frameworks.

This study uniquely contributes to the literature by applying a validated psychometric tool (DASS-21) validated in Arabic by Alharbi and Osman [17] to assess the trajectory of depression, anxiety, and stress levels surrounding cadaveric exposure among medical students of Higher Diploma of Pre-Clinical Sciences (HDPCS) programme. Furthermore, it extends global discourse by incorporating cultural perspectives and comparing findings to established international data (e.g.) [4,6].

This study therefore integrates psychological, educational, and cultural dimensions to provide a more coherent framework for understanding early emotional responses to cadaveric exposure. The integration of Lazarus and Folkman’s stress appraisal model supports the interpretation of pre-exposure anxiety and post-exposure adaptation as part of cognitive reappraisal and coping processes. Additionally, the research team’s interdisciplinary composition—combining expertise from anatomy and psychology—ensures reflexive awareness in data interpretation and strengthens methodological rigour in linking emotional states to learning outcomes. (reviewer 2—Comment 1).

### Research question

What are the levels of emotional distress (i.e. depression, anxiety, and stress) experienced by first-year medical students at Medical Gulf University before and after their initial exposure to cadaveric dissection?

### Objectives

This study aims to assess the levels of psychological distress—specifically depression, anxiety, and stress—among medical students at Gulf Medical University before and after their initial exposure to cadaver dissection in the dissection room.

### Hypotheses

**H1**: First-year medical students report elevated levels of emotional distress (depression, anxiety, and stress) before their initial dissection room exposure, which tend to decrease following the experience.

**H2**: There is a statistically significant difference in emotional distress levels (depression, anxiety, and stress) between the pre- and post-dissection session.

### Methodology

Study Design: This quantitative, comparative, cross-sectional study explores emotional distress among first-year medical students before and after their first exposure to the dissection session.

At Gulf Medical University, anatomy teaching begins early in the first year within the Higher Diploma in Pre-Clinical Sciences (HDPCS) programme. Students are first exposed to cadaveric material during their initial semester as part of the ‘Human Anatomy and Embryology’ course. The DASS-21 questionnaire was administered immediately before and after the first full-body dissection session conducted under faculty supervision in the gross anatomy laboratory. Prior to this exposure, students received a brief orientation session that outlined dissection ethics, safety protocols, and professional conduct in the dissection room. (Reviewer 2 Comment 3).

Participants: The study included 80 first-year medical students selected from the Higher Diploma in Pre-Clinical Sciences (HDPCS)programme at the College of Medicine in Ajman. The participants’ ages ranged from 17 to 18 years, with a mean age of 17.7 years and a standard deviation of 0.6.

The participating students were enroled in the Higher Diploma in Pre-Clinical Sciences (HDPCS) programme, representing diverse cultural backgrounds, including Emirati, Indian, Pakistani, Egyptian, and other Arab nationalities. Most participants had completed high school education within the UAE and were exposed for the first time to formal anatomy teaching through cadaveric dissection. Culturally, the group reflected a mix of religious and social values that emphasise respect for human remains and emotional restraint in academic settings. Such diversity provided an important context for understanding how cultural and educational backgrounds shape emotional reactions and coping behaviours during anatomy training.

Study Instrument: The Depression Anxiety Stress Scales-21 (DASS-21) is a widely recognised self-report tool developed by Lovibond and Lovibond [18] to measure the three related emotional states of depression, anxiety, and stress. It is the short form of the original 42-item DASS and contains 21 items, equally divided into three subscales: Depression, Anxiety, and Stress. Each item is rated on a 4-point Likert scale ranging from 0 (‘Did not apply to me at all’) to 3 (‘Applied to me very much or most of the time’), reflecting the respondent’s experience over the past week. The total score for each subscale is calculated by summing the responses to the relevant 7 items and then multiplying the total by 2, which ensures equivalence with the DASS-42 scoring system. The Depression subscale captures symptoms such as sadness, lack of motivation, and self-deprecation; the Anxiety subscale measures physiological symptoms of fear, panic, and nervous arousal; while the Stress subscale reflects chronic tension, irritability, and relaxation difficulties.

Each of the three subscales can be used independently to measure a specific emotional domain without needing to administer or analyse the full scale, depending on the aim of the assessment. This independent use of subscale totals is supported by research demonstrating that the three constructs—though related—are statistically distinct, with each subscale showing high internal consistency and validity when analysed on its own [19,20].

The assessment tool (the DASS-221) has been approved in languages and cultural settings such as Arabic, Spanish, Chinese, Persian, Turkish, Thai, and Amharic. For example, the Arabic edition demonstrated good reliability within university students, with alpha coefficients of 0.88, 0.84, and 0.87 for depression, anxiety, and stress, respectively [21]. The Spanish edition has demonstrated dependability and factorial credibility (according to [22]. Additionally, a recent Thai validation study in 2023 indicated alpha coefficients of 0.82 for depression, 0.78 for anxiety, and 0.69 for stress [23]. On the other hand, the latest Spanish version validation indicated omega coefficients of 0.90 for depression, 0.84 for anxiety, and 0.90 for stress [24].

The Arabic DASS-21 was evaluated for its psychometric properties in a large-scale study involving 1,235 participants from Saudi Arabia, representing varied demographics. The study found that the scale demonstrated excellent internal consistency, with a Cronbach’s alpha of 0.94 for the total scale, and good reliability for the subscales: depression (*α* = 0.87), anxiety (*α* = 0.84), and stress (*α* = 0.86). The confirmatory factor analysis supported the acceptability of the three-factor model, although some items showed cross-loadings on different factors compared to the original structure, suggesting areas for refinement [25].

This highlights the dependability, validity, and versatility of DASS 21 across demographics and research environments.

The DASS-21 questionnaire was administered in-person immediately before and after the dissection session. Students completed the survey anonymously without faculty present in the laboratory to reduce social desirability bias. No identifying information was collected.

The research team consisted of anatomy and psychology faculty members involved in teaching the participating cohort. To minimise potential power dynamics, data collection was anonymous, and students were assured that participation or responses would not influence academic evaluation. The interdisciplinary nature of the research team supported balanced interpretation by integrating both educational and psychological perspectives.

### Statistical methods

Mean, standard deviation and *t*-test were computed. SPSS was used in these analyses.

## Results

This section presents the empirical results of the study, summarising the main findings based on the collected data.

**Figure 1.**
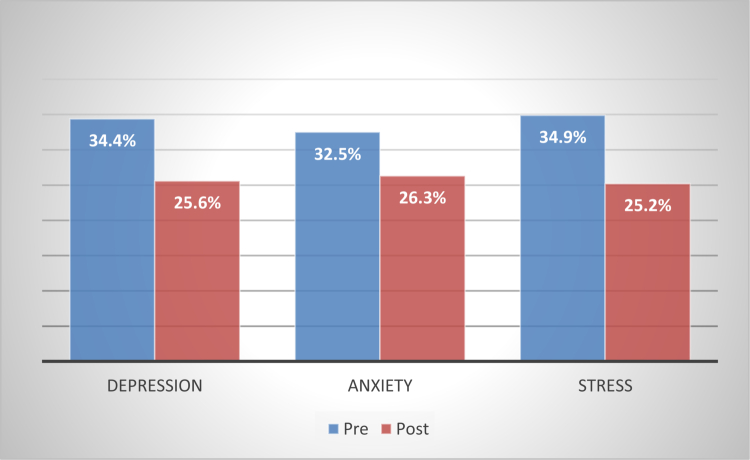
Percentage change in depression, anxiety, and stress scores before and after first cadaver dissection exposure among first-year medical students (*N* = 80).

To test the first hypothesis, the percentage of emotional distress levels (depression, anxiety, and stress) was calculated before and after exposure to the dissection room in the same session. Table 1 and Figure 1 illustrate the comparative results.

**Table 1.** Emotional distress scores before and after exposure to the dissection room (*N* = 80).

Variable	Pre-exposure (%)	Post-exposure (%)	Decrease (%)
Depression	34.4%	25.6%	↓ 8.8%
Anxiety	32.5%	26.3%	↓ 6.2%
Stress	34.9%	25.2%	↓ 9.7%

As illustrated in Table 1, the emotional distress levels among first-year medical students were elevated before their exposure to the dissection room, namely in stress (34.9%), depression (34.4%), and anxiety (32.5%). Following the exposure, there was a noticeable decline in all three domains: stress reduced to 25.2%, depression to 25.6%, and anxiety to 26.3%. These findings suggest that although students initially experience heightened emotional distress, this distress tends to lessen after their initial encounter with the dissection room environment. Therefore, the first hypothesis was fully verified.

To examine the second hypothesis, (H2: There is a statistically significant difference in emotional distress levels (depression, anxiety, and stress) between the pre- and post-dissection session), one-way Analysis of Variance (ANOVA) was conducted to determine the differences between pre- and post-dissection scores for depression, anxiety, and stress (see Table 2).

**Table 2.** One-way ANOVA results for depression.

Source	Sum of squares	Df	Mean square	F	Sig.
Between Groups	547.60	1	547.60	3.89	.050
Within Groups	22234.80	158	140.72		
Total	22782.40	159			

The F-value for depression in Table 2 (F = 3.891) is statistically significant at the 0.05 level, indicating that there is a significant difference between the pre- and post-exposure. To specify the direction of the difference, a paired samples t-test was calculated to determine this direction. The results are presented in Table 3.

**Table 3.** Paired samples t-test for depression scores pre- and post-exposure.

Measure	N	Mean	Std. deviation	Std. error mean	*t*	*p* (2-tailed)	R²
Depression (Pre)	80	14.45	12.29	1.37			
Depression (Post)	80	10.75	11.42	1.28	1.973	0.050	.54

To explore the direction of the significant variance identified through the ANOVA, a paired samples t-test was conducted comparing depression scores before and after exposure. Table 3 shows a decrease in mean depression scores from pre- to post-exposure; however, this difference did not reach clear statistical significance [The t test is significant at 0.05 level].

**Table 4.** One-way ANOVA results for anxiety.

Source	Sum of squares	Df	Mean square	F	Sig.
Between groups	270.40	1	270.40	2.09	.150
Within groups	20422.00	158	129.25		
Total	20692.40	159			

The results from the ANOVA (Table 4) for anxiety scores did not show a statistically significant difference between pre- and post-dissection groups (F = 2.09, *p* = .150).

The one-way ANOVA conducted to examine differences in stress scores between the pre- and post-exposure groups (Table 5) revealed a statistically significant difference, indicating a meaningful variation between the two conditions.

**Table 5.** One-way ANOVA results for stress.

Source	Sum of squares	df	Mean square	F	Sig.
Between groups	664.22	1	664.22	4.76	.030
Within groups	22007.75	158	139.29		
Total	22671.97	159			

The paired samples t-test conducted on behalf of the 80 participants (Table 6) revealed a statistically significant difference in stress scores before and after exposure.

**Table 6.** Paired samples t-Test for stress scores pre- and post-exposure.

Measure	N	Mean	Std. deviation	Std. error mean	*t*	*p* (2-tailed)	R²
Stress (Pre)	80	14.65	12.11	1.35			
Stress (Post)	80	10.58	11.49	1.28	2.184	.030	.569

## Discussion

The results of the first hypothesis revealed that stress levels among students were higher than both depression and anxiety levels before their first exposure to the dissection room. Anxiety, in contrast, was found not to be statistically significant. This result of declining anxiety aligns with the result of a recent study about the degree of satisfaction and anxiety among first-year students in medicine, occupational therapy, speech therapy and nursing programmes in Spain. It shows this decline in anxiety throughout the semester, with medical students scoring the highest. Nonetheless, students were satisfied and 96.8% of them suggested that the practices be kept for future courses. Yet, the experience can cause stressful reactions that need to be addressed with advanced preparation and coping mechanisms, particularly among medical and nursing students [6]. The prominence of stress before cadaver exposure can be explained by the unique psychological burden associated with the anticipation of the dissection experience. Several studies have noted that dissection may provoke fear and apprehension in medical students as it represents their initial real-life encounter with human mortality in an educational setting [5,26].

When comparing the current findings to studies conducted in similar regional contexts, comparable patterns were observed. In Oman, Abu-Hijleh et al. [4] reported that 38% of students experienced recurring visual images of cadavers, while 22.5% reported appetite loss after dissection exposure. In Jordan, Bataineh et al. [14] found 28.8% of students reported fear and 19.3% palpitations—levels closely aligned with those observed among the present UAE cohort. These similarities suggest that students in Middle Eastern medical schools share common psychosocial stressors associated with the strong ethical and cultural meanings attached to human dissection. However, unlike some regional institutions that reported sustained anxiety levels weeks after exposure, our results show a more rapid emotional adaptation post-dissection, which may be attributed to structured faculty guidance and peer support implemented at Gulf Medical University. Reviewer 1 (Comment 2).

Furthermore, stress in this context is often compounded by a combination of academic pressure and uncertainty about personal emotional responses to cadaver handling [27]. Stress, according to Lazarus and Folkman’s transactional theory, results from the cognitive appraisal of a situation as taxing or exceeding the individual's coping resources [28]. Thus, it is plausible that students initially interpret the upcoming dissection experience as threatening, given its novelty and the perceived emotional and ethical complexities [29].

Moreover, previous findings suggest that stress is often elevated in early medical training, particularly before high-stakes or emotionally charged experiences [16,30]. Students may fear failure, judgement from peers or instructors, or worry about violating cultural or religious taboos related to human remains [31]. The heightened stress response, therefore, reflects not only an emotional reaction but also a complex socio-cultural and cognitive appraisal of the dissection process.

Additionally, looking into the topic from a different perspective highlights the importance of the coping mechanisms in interpreting the results. Students frequently use techniques like staying in groups for psychological support (58%) and concentrating on the task at hand (62%) to deal with these psychological difficulties [4,14,32]. Others manipulate cognitive coping such as rationalisation and intellectualisation [14].

In addition, emotional and physical coping mechanisms are used as well as colleagues’ help, to mitigate the negative effects. Colleagues' support constitutes another affordable, feasible, and effective way to counteract stress and anxiety resulting from the first encounter with the dissection room. For instance, on the first day of gross anatomy dissection, first-year medical students found that third-year medical students' help was a simple, affordable, and instructive way to reduce their physical and mental stress. In comparison with students who were not accompanied by more senior students, medical students in the first year (MS1) showed significant differences in both physical and emotional reactions. MS1 had significantly fewer physical reactions (64% vs. 88%), reporting lower levels of anxiety (23% vs. 48%), headache (14% vs. 36%), disgust (9% vs. 20%), feeling light-headed (11% vs. 24%), and reaction to the smell of the cadaver and laboratory (8% vs. 52%) [13].

Concerning the second hypothesis, the statistical analysis showed significant differences in depression scores, particularly higher levels in the pre-exposure phase. This outcome indicates that students entering the dissection room are already experiencing heightened depressive symptoms, which tend to decrease afterward. These findings align with earlier research suggesting that anticipatory psychological distress is prevalent among students before dissection, and the act of exposure itself may serve to normalise the experience and reduce its emotional burden [5,27,33].

One explanation for the elevated depression scores before exposure may lie in the emotional weight of dissection being perceived as morally or existentially challenging [30]. Many students face conflicting feelings regarding respect for the dead, discomfort with bodily remains, and fear of emotional inadequacy in coping [4,34]. This may result in symptoms such as low mood, decreased motivation, or even feelings of guilt, commonly associated with depressive states [11].

However, post-exposure, the depressive symptoms showed a measurable reduction. This may be due to students’ cognitive restructuring as they become more desensitised or develop more professional coping mechanisms [35]. Additionally, engaging directly with the cadaver may facilitate the development of professional identity and foster adaptive learning attitudes, thus alleviating some of the emotional weight carried beforehand [36,37].

As for stress, although it remained high in both phases, it was also significantly higher before the dissection. This pattern has been attributed to the psychological load that precedes actual participation in emotionally sensitive medical procedures. The work of Chia et al. [5] and Nnaka et al. [1] supports the interpretation that the unfamiliarity of the dissection room, coupled with sensory stimuli (such as smell and visual exposure), contributes to anticipatory tension. Once students engage with the task, these stimuli become predictable, and anxiety may decline, allowing stress to be processed and managed more effectively [13].

In addition, though such psychological reaction was found to be worldwide, anyone going through these experiences is probably afraid of being stigmatised and declared unfit, so students keep the information to themselves. This is accompanied by the stereotypical belief that a person is not cut out for the medical profession if they cannot handle these testing situations. Therefore, if a student exhibits no emotions at all during the dissection practical, coping is deemed successful. It is inconsistent with entirely objective, scientific professional behaviour to act in any other way [30].

Future research at GMU may adopt a longitudinal comparative design to assess students’ emotional responses before and after the implementation of these proposed institutional measures—such as pre-dissection orientation sessions, reflective discussions, and memorial ceremonies. By comparing the emotional distress levels of current cohorts with those of subsequent ones after introducing these interventions, it would be possible to determine their long-term effectiveness in reducing anxiety and stress, enhancing resilience, and promoting professional identity formation among medical students [35,36]. (Reviewer 1 -Comment 3).

A distinctive contribution of this study lies in its Gulf-region context, where cultural and religious attitudes toward death and human remains influence emotional expression and coping strategies. Unlike Western cohorts, students in this study operate within sociocultural frameworks that value composure and respect for the deceased, which may initially heighten anticipatory anxiety but facilitate rapid emotional recalibration once the experience is contextualised as a professional duty [4,36]. (Reviewer 2—Comment 6).

Based on the present findings, future research at GMU may adopt a longitudinal comparative design to evaluate the impact of structured preparatory and reflective interventions on emotional adaptation to cadaveric dissection. Specifically, subsequent cohorts can be introduced to pre-dissection orientation workshops, guided reflective discussions, and a donor appreciation or memorial ceremony. Emotional distress scores of these future cohorts can then be compared to the current baseline data to determine whether such psychological and educational support strategies reduce anticipatory anxiety and enhance professional identity formation. This approach would allow clearer insight into both the short-term and sustained effects of institutional coping interventions within this cultural context (reviewer 1).

### Limitations

While this study offers important insights into the emotional responses of medical students during their first cadaver dissection experience, a few limitations must be acknowledged. First, the research was conducted within a single academic institution, involving a relatively uniform group of first-year students enroled in the HDPCS programme. As such, the findings may not be fully generalised to students from different educational backgrounds or cultural contexts.

Second, although DASS-21 is a validated and widely used tool, it may not fully capture the complexity of students’ emotional experiences in this unique setting. Important factors such as previous exposure to stress or trauma, personal coping styles, and individual personality traits were not assessed, though they may have influenced the outcomes. Third, the study followed a cross-sectional comparative design, which limits the ability to conclude long-term psychological impact. A longitudinal approach could offer a clearer picture of how emotional responses develop or diminish over time.

Moreover, because emotional responses were self-reported, some reactions may have been under expressed due to cultural norms that encourage emotional restraint in academic and clinical environments.

## Conclusion

In sum, the present findings reinforce existing literature that emphasises the value of structured psychological preparedness and emotional support for students entering dissection labs [32]. Reducing the emotional burden before exposure could be key to enhancing student adaptation and well-being in anatomy education.

To ameliorate the emotional reactions among medical students in the present context, we suggest developing a brief audio-visual programme to familiarise students to this course before the beginning of the actual practice to a full cadaver dissection. Other helpful techniques to lessen stress, anxiety, and depression, such as peer support groups, faculty mentoring, preparatory workshops, gradual exposure, and virtual dissection, are also recommended.

Additional institutional measures are strongly recommended to mitigate the psychological impact of cadaver exposure. Prior orientation sessions that include open class discussions about death, professionalism, and emotional responses can prepare students for their first encounter. Reflective sessions following the dissection can further normalise emotional expression and foster resilience. Moreover, organising a memorial or gratitude ceremony led by students to honour body donors has proven effective in several medical schools [35,36]. Such rituals help students transform anxiety into gratitude, reinforce ethical awareness, and cultivate empathy—critical competencies in medical education. Incorporating these practices into the curriculum could promote both emotional regulation and professional identity formation among medical students at GMU.

In alignment with best practices demonstrated in international medical schools, we additionally recommend that Gulf Medical University consider the integration of a student-led donor appreciation or memorial ceremony as part of the anatomy curriculum. Such ceremonies, commonly held to honour the individuals who donated their bodies to medical education, have been shown to foster gratitude, ethical awareness, empathy, and emotional adjustment among students. In the present cultural context, where respect for human remains and emotional composure are highly valued, a memorial ceremony would allow students to transform initial distress into professional reverence, supporting both emotional regulation and compassionate identity development within the medical profession. **(Reviewer 1).**

## Data Availability

The datasets generated and/or analysed during the current study are available from the corresponding author on reasonable request.
